# The RRM of the kRNA-editing protein TbRGG2 uses multiple surfaces to bind and remodel RNA

**DOI:** 10.1093/nar/gky1259

**Published:** 2018-12-14

**Authors:** Brady Travis, Porsha L R Shaw, Bei Liu, Krishna Ravindra, Hadley Iliff, Hashim M Al-Hashimi, Maria A Schumacher

**Affiliations:** 1 Department of Biochemistry, Duke University School of Medicine, Durham, NC 27710, USA; 2 Department of Chemistry, Duke University School of Medicine, Durham, NC 27710, USA

## Abstract

Kinetoplastid RNA (kRNA) editing takes place in the mitochondria of kinetoplastid protists and creates translatable mRNAs by uridine insertion/deletion. Extensively edited (pan-edited) transcripts contain quadruplex forming guanine stretches, which must be remodeled to promote uridine insertion/deletion. Here we show that the RRM domain of the essential kRNA-editing factor TbRGG2 binds poly(G) and poly(U) RNA and can unfold both. A region C-terminal to the RRM mediates TbRGG2 dimerization, enhancing RNA binding. A RRM-U4 RNA structure reveals a unique RNA-binding mechanism in which the two RRMs of the dimer employ aromatic residues outside the canonical RRM RNA-binding motifs to encase and wrench open the RNA, while backbone atoms specify the uridine bases. Notably, poly(G) RNA is bound via a different binding surface. Thus, these data indicate that TbRGG2 RRM can bind and remodel several RNA substrates suggesting how it might play multiple roles in the kRNA editing process.

## INTRODUCTION

Kinetoplastids are a group of flagellated protists that include the parasitic protozoa, *Trypanosoma brucei, T. cruzi* and *Leishmania* spp., which are the causative agents of African sleeping sickness, Chagas disease and leishmaniasis (1). Kinetoplastids are named after their unusual mitochondrial DNA, called the kinetoplast (2). Kinetoplastid protists employ several unusual biological processes that represent attractive targets for drug development, perhaps the most notable of which is kinetoplastid RNA editing (kRNA editing). This process, which takes place in the mitochondria, involves the specific insertion and/or deletion of uridine nucleotides and is necessary to generate translatable mitochondrial mRNAs from nonfunctional cryptogene derived mRNAs (3–6). In *T. brucei* 12 of the 18 mitochondrial mRNA transcripts require editing to produce functional mRNAs. The extent of editing among the mRNAs varies. Minimally edited transcripts require the addition or deletion of only a few uridines, while pan-edited transcripts, which include nine of the mRNAs, necessitate the addition and deletion of hundreds of uridine nucleotides (3–7).

The kRNA editing process is catalyzed by large editosome complexes (3–6,8) and directed by small RNAs called guide RNAs (gRNAs), which dictate where and how many uridines are added and removed (9–13). However, editosomes are not sufficient for full editing; the process is highly dynamic, requiring interactions between multiple gRNA and pre-mRNA molecules. These dynamic RNA-RNA interactions are faciliated by so-called kRNA editing accessory factors. Recent analyses of pan-edited pre-mRNAs by the Göringer lab also revealed that they contain multiple guanine-rich (G-rich) stretches prone to higher order RNA structure formation that must be resolved to permit the multiple addition/deletion steps, which proceeds with a 3′ to 5′ directionality (14–15). Thus, uridine and guanine repeat elements are an apparent characteristic of kRNA editing. Consistent with the complexity of the kRNA editing process, numerous accessory factors have been identified and shown to facilitate this process (16–33). kRNA accessory proteins that have been characterized to date include RBP16, MRP1/MRP2, p22, the RGG proteins and more recently, a large multiprotein complex called the RNA editing substrate binding complex (RESC), which is also known as the mitochondrial RNA binding complex 1 (MRB1) (18–25). The RESC complex appears to be heterogenenous but contains several accessory factors. Within the RESC are two subcomplexes called the guide RNA binding complex (GRBC also known as the MRB1 core) and a more dynamic complex termed the RNA editing mediator complex (REMC) (19–25).

A notable characteristic of kRNA editing accessory proteins is that they typically show limited or no sequence homology to known proteins, making a detailed molecular understanding of these proteins challenging. Indeed, to date, only a handful of accessory factors have been studied at the structural level. These include the MRP1/MRP2 complex, p22 and MRB1590 (16,27,32). MRB1590 and MRP1/MRP2 transiently associate with the RESC complex and both interact directly with RNA to affect kRNA editing dynamics (27,32). The trimeric p22 protein displays structural homology to the p32 RNA splicing factor (16). However, p22 does not bind RNA but rather appears to mediate protein-protein interactions with other accessory proteins. Studies indicate that these factors are required for editing a limited set of mRNA transcripts; MRP1/MRP2 primarily effects editing of the cytochrome *c* (CYb) transcript (33), MRB1590, subunit 6 of the ATPase (A6) transcript (32) and p22, the cytochrome oxidase subunit II (COXII) transcript (16). Unlike these editing accessory proteins, the *T. brucei*RGG2 (TbRGG2) protein is required for editing all nine pan-edited mRNAs (28–30). Pan-editing involves multiple insertion and deletion steps directed by several gRNAs within a given mRNA. In this process uridine nucleotides are inserted and removed into the pre-mRNA as poly(U) blocks. For example, pan-editing of the mRNA encoding the ND7 subunit of the *T. brucei* NADH dehydrogenase requires the addition and deletion of 553 and 89 uridines, respectively (13) (Supplemental Figure S1). Studies in the Read laboratory showed that TbRGG2 depletion significantly affects the progression of the editing process (28). *T. brucei* displays differentiation dependent mechanisms of metabolism depending on whether it is in the insect (tsetse fly) procyclic form (PF) or the stage that exists in mammalian hosts, called the bloodstream form (BF). TbRGG2 was shown to be essential for growth in both PF and BF forms of the parasite (29).

TbRGG2 is a 330 residue protein and contains two main domains, an N-terminal glycine (Gly)-rich region composed of GWG and RG repeats and a C-terminal RNA recognition motif (RRM) (28–30). Gly-rich motifs are found in multiple RNA binding proteins and are typically disordered until bound to RNA (34–35). RRM domains consist of ∼70–80 residues and contain so-called RNP motifs that are involved in nucleic acid binding (36–37). TbRGG2 binds RNA and modulates RNA structure and these functionalities have been shown to be to be essential for its role in kRNA editing (28–30). A separation of function analysis of TbRGG2 domains, examining its binding to mRNA fragments, suggested that the protein has both RNA annealing and melting activities with these activities being assigned to the Gly-rich and RRM regions, respectively (30). Thus, TbRGG2 has been established as an essential kRNA editing factor. However, understanding the molecular mechanisms by which TbRGG2 mediates its functions requires structural information, which has to date been lacking. Here, we carry out a detailed analysis of the TbRGG2 C-terminal region that encompasses its RRM domain. RNA binding studies show that the TbRGG2 RRM binds both U and G-rich RNA with high affinity and can unfold both RNA elements. Notably, an RRM-U4 structure shows that although the RRM domain harbors a typical RRM fold it binds poly(U) RNA using a mechanism distinct from the canonical RRM binding mode. Moreover, the TbRGG2 RRM binds RNA as a dimer and data reveal that a region directly C-terminal to the RRM, harboring a predicted coiled-coil, mediates dimerization. We also show that the TbRGG2 RRM binds poly(G) RNA using residues distinct from those used to bind poly(U), revealing the RRM as a multimodal RNA binding region.

## MATERIALS AND METHODS

### Expression and purification of *T. brucei* TbRGG2 RRM and RRM-Cterm

The DNA region encoding the TbRGG2 RRM domain (residues 203–269) and the TbRGG2 RRM-Cterm (residues 203–320) were PCR amplified and cloned into pET15b between the BamHI and NdeI restriction sites. Each expression plasmid was then transformed into *Escherichia coli* C41(DE3) cells. Protein expression of each was induced by addition of isopropyl β-d-1-thiogalactopyranoside (IPTG) to a final concentration of 0.5 mM for 4 hours (h) at 37°C when the cells had reached an *A*_600_ of 0.4–0.6. The expressed proteins were found in the soluble fraction and were purified using nickel-nitrilotriacetic acid (Ni-NTA) column chromatography. The His6 tags were cleaved using thrombin cleavage capture kits (Qiagen). The His-tag free proteins were further purified by Superdex 75 size exclusion column chromatography. RRM and RRM-Cterm mutants were made using the Quikchange mutagenesis protocol and expressed and purified as per the WT proteins.

### Crystallization and structure determination of the TbRGG2 RRM

Crystals of the WT RRM domain were obtained via hanging drop vapor diffusion at room temperature (rt) by mixing protein at 10 mg/ml 1:1 with a crystallization reagent consisting of 0.1 M 4-(2-hydroxyethyl)-1-piperazineethanesulfonic acid (HEPES) pH 7.5 and 1.4 M trisodium citrate. Crystals took several weeks to grow to final size and were cryo-protected straight from the drop. Data were collected to 1.8 Å at the Advanced Light Source (ALS) beamline 8.3.1, processed with MOSFLM and scaled with SCALA. The crystals take the tetragonal space group, *P*4_1_2_1_2 (Table 1). The fact that there was no RRM structure with high homology to the TbRGG2 RRM and the large number of subunits (>3) in the crystallographic asymmetric unit (ASU) predicted by the Matthews coefficient, was consistent with the failure of molecular replacement (MR) using previously solved RRM domains as search models. Hence a RRM(L209M) mutant protein was generated to use in Selenomethionine (SeMet) single wavelength anomalous diffraction (SAD) or multiple wavelength anomalous diffraction (MAD) experiments to obtain phases, as the WT RRM does not contain methionines. Native (non SeMet) RRM(L209M) protein produced a different crystal form than the WT RRM. These crystals were obtained by the hanging drop vapor diffusion method using protein at 10 mg/ml and mixing it 1:1 with either 0.1 M Tris–HCl pH 7.0 and 1.2 M trisodium citrate or 0.1 M Tris–HCl pH 7.5 and 1 M magnesium sulphate. The crystals were cryo-preserved by dipping them in a solution consisting of the crystallization reagent with 15% (v/v) glycerol for 1–2 s before placement in the cryo-stream. Data were collected to 1.8 Å at ALS beamline 8.3.1, processed with MOSFLM and scaled with SCALA. The RRM(L209M) crystals contain one subunit in the ASU, suggesting the possibility that MR could be successfully employed to solve the structure. Several RRM structures were used in MR attempts and indeed a correct solution was obtained using residues 17–86 of the SRSF1 RRM domain (PDB code 2M8D) as a search model. Hence, SeMet phasing was not necessary to solve the structure. After multiple cycles of rebuilding, including replacement of the SRSF1 side chains with those from TbRGG2 in Coot and refinement using Phenix (38–39), the model converged to final *R*_work_/*R*_free_ values of 15.1%/18.7%. The final model includes all residues of the RRM and 93 water molecules. See Table 1 for final data collection and refinement statistics. The RRM(L209M) structure, after mutating the Met209 residue to leucine, was then used in MR to solve the WT *P*4_1_2_1_2 RRM structure with Phaser. After multiple rounds of refinement and model optimization in Coot, the structure converged to final *R*_work_/*R*_free_ values of 16.5%/19.7% to 1.8 Å resolution (Table 1). The final model includes all residues of the RRM for the four subunits in the ASU and 326 water molecules.

**Table 1.** Data collection and refinement statistics for *T. brucei* TbRGG2 RRM and TbRGG2 RRM-RNA complex

	TbRGG2 RRM(L209M)	WT TbRGG2 RRM	WT TbRGG2 RRM-U4 RNA
**Data collection**			
Pdb code	6E4N	6E4O	6E4P
Space group	*R*3	*P*4_1_2_1_2	*P*2_1_
Cell dimensions			
*a, b, c* (Å)	59.8, 59.8, 57.5	72.1,72.1, 118.7	49.1,126.3, 49.2
α, β, γ (°)	90.0, 90.0, 120.0	90.0, 90.0, 90.0	90.0, 118.6, 90.0
Resolution (Å)	19.26–1.80	38.64–1.80	35.66–1.95
Total reflections (#)	23 430	74 490	146 384
Unique reflections (#)	7045	28 448	38 345
*R*_sym_	0.070 (0.249)*	0.050 (0.212)	0.066 (0.351)
*R*_pim_	0.044 (0.161)	0.035 (0.208)	0.040 (0.280)
CC(1/2)	0.994 (0.960)	0.997 (0.977)	0.998 (0.990)
Completeness (%)	96.0 (91.3)	96.0 (80.3)	99.8 (99.0)
Redundancy	3.2 (1.7)	2.6 (1.4)	3.8 (3.5)
I/σI	11.3 (4.2)	12.5 (2.3)	17.8 (2.5)
Resolution (Å)	19.26–1.80	38.64–1.80	35.66–1.95
*R*_work_/*R*_free_ (%)	15.1/18.7	16.5/19.7	18.1/22.9
R.M.S. deviations			
Bond lengths (Å)	0.004	0.006	0.005
Bond angles (°)	0.763	0.890	0.848
Ramachandran analyses			
Most favored (%)	100.0	99.4	99.7
Disallowed (%)	0.0	0.0	0.0

*Values in parentheses are for highest-resolution shell.

Crystals of the WT TbRGG2 RRM domain complexed with a 4-mer poly(U) RNA (herein termed U4) were obtained by mixing protein (at 10 mg/ml) and RNA (in a 1:1 molar ratio) with a crystallization solution 1:1 consisting of 1.2–1.3 M sodium citrate and 0.1 M HEPES pH 7.0. X-ray intensity data was collected at the Advanced Photon Source beamline 22-ID and processed with HKL-2000 (40). The structure was solved by MR using the apo WT RRM domain as a search model in Phaser. The structure was manually rebuilt in Coot and refined in Phenix (38–39). The final model includes 9 copies of the RRM domain, three uridines are visible of each U4 RNA and 314 water molecules (Table 1).

### Fluorescence Polarization (FP) experiments

FP experiments were performed using a PanVera Beacon 2000 FP system at 25°C. 5′-Fluoresceinated 20-mer poly(A) (A20), 20-mer poly(C) (C20), 20-mer poly(G) (G20), 20-mer poly(U) (U20), 7-deazaguanine and *N-*1-methylguanine oligonucleotides were used for FP experiments to analyze RNA binding by WT RRM, WT RRM plus C-terminal domain (RRM-Cterm) and mutant RRM and RRM-Cterm proteins. For the experiments, purified proteins were titrated into 0.990 mL buffer composed of 20 mM Tris–HCl pH 7.5, 5% (v/v) glycerol and 50 mM NaCl, 20 mM and containing 1 nM (final concentration) fluorescently labelled RNA. Data were fit using Logger Pro. The binding curves were normalized (normalized mP) using the equation: (mP – mP_0_)/(mP_max_ – mP_0_) × 100, where mP_0_ is the minimal mP for the binding assay and mP_max_ is the maximum mP value. Each binding curve is a representative curve from at least three technical replicates.

### Size Exclusion Chromatography (SEC) analysis of TbRGG2 RRM-Cterm

For the SEC analyses of the RRM and RRM-Cterm, the proteins were concentrated to 10 mg/ml and each were loaded onto a Superdex 75 size exclusion column and eluted with a buffer containing 20 mM Tris–HCl pH 7.5, 150 mM NaCl, 5% (v/v) glycerol, and 1 mM DTT. The elution volumes were compared to a series of protein standards to determine the molecular weights. The standards used were cytochrome c oxidase (12.4 kDa), carbonic anhydrase (29 kDa) and albumin (66 kDa). For the SEC experiment with protein and RNA, purified TbRGG2 RRM-Cterm at 16 mg/ml was mixed with equimolar U20 RNA. The protein/RNA mixture was loaded onto a Superdex 75 size exclusion column and eluted in the same buffer as used for the protein alone. Two peaks were obtained from the size exclusion column, one corresponding to TbRGG2 RRM-Cterm with U20 RNA and one corresponding to excess U20.

### RNA sample preparation

The quadruplex-forming 5′-UAGGGUUAGGGU-3′ oligonucleotide and modified derivatives of this RNA were synthesized using a MerMade 6 Oligo Synthesizer employing 2′-tBDSilyl protected phosphoramidite (ChemGenes) on 1 μM standard synthesis columns (1000 Å) (BioAutomation). Fluorescein and 7-deazaguanine phosphoramdites were purchased from Glen Research and ChemeGenes, respectively. The synthesized oligonucleotides were cleaved from the 1 μM column using 1 ml ammonia methylamine followed by 2 h incubation at rt for base deprotection. The solutions were then air-dried and dissolved in 115 μl DMSO, 60 μl TEA and 75 μl TEA-3HF for 2′O deprotection followed by 2.5 h incubation at 65°C. Samples were purified using Glen-Pak RNA cartridges (Glen Research Corporation) (http://www.glenresearch.com). Samples were ethanol precipitated, air dried, and dissolved in water. All other RNA oligonucleotides used for crystallization and RNA-binding assays were purchased from Dharmacon. To assay binding by the 7-deazaguanine modified RNA all guanines in the quadruplex-forming sequence, 5′-UAGGGUUAGGGU-3′, were replaced with 7-deazaguanine while for the m1G modified sequence, only the first three guanines were replaced with m1G. The purities of the modified and unmodified oligonucleotides were verified by NMR as described (41).

### 1H-NMR spectroscopy

NMR experiments were performed on a 600 MHz Bruker NMR spectrometer equipped with an HCN cryogenic probe. Data were processed and analyzed using NMRpipe (42). The NMR buffer used was: 15 mM sodium phosphate pH 7.5 and 50 mM KCl. 2D CD SOFAST-HMQC aromatic data were obtained and analyzed as previously described (43).

## RESULTS

### TbRGG2 RRM RNA binding specificity and structure

**Figure 1.**
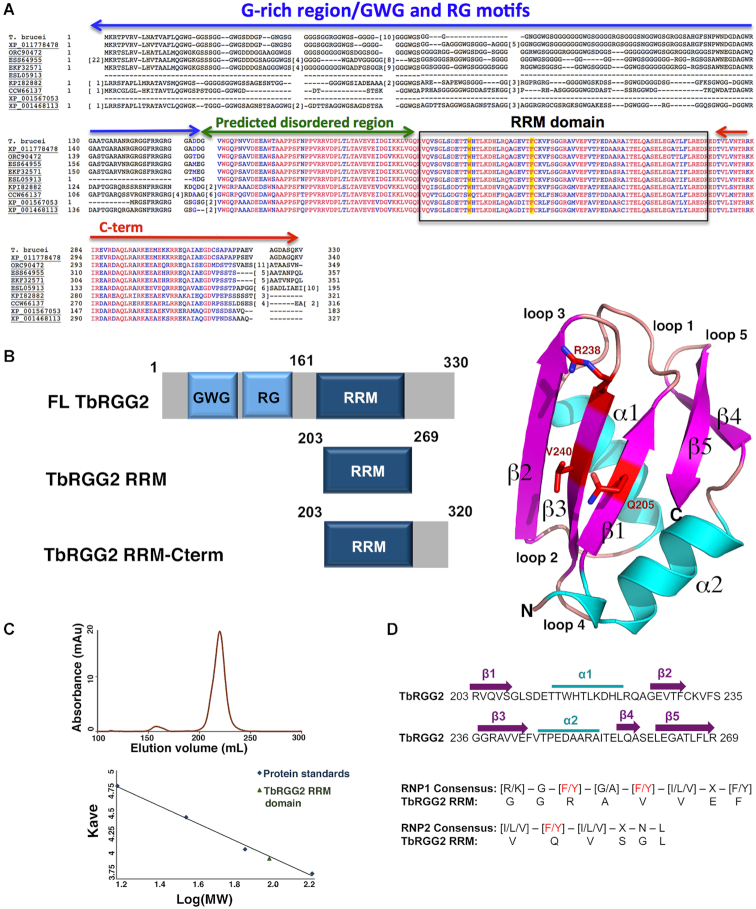
*T. brucei* TbRGG2 RRM structure. (**A**) Multiple sequence alignment of TbRGG2 orthologs with conserved domains labeled over the sequence. The codes represent RGG2 proteins from the specified protists. XP_011778478, *Trypanosoma gambiense* DAL972; ORC90472, *Trypanosoma theileri*: ESS64955, *Trypanosoma cruzi* Dm28c; EKF32571, *Trypanosoma cruzi marinkellei*; ESL05913, *Trypanosoma rangeli* SC58; KPI82882, *Leptomonas seymouri*; CCW66137, *Phytomonas* sp. Isolate Hart1; XP_001567053, *Leishmania braziliensis* MHOM/BR/75/M2904; XP_001468113, *Leishmania infatum* JPCM5. Boxed is the RRM domain and highlighted in yellow are residues shown to be required for poly(U) binding by the TbRGG2 RRM domain. Red and blue residues represent completely and highly conserved residues, respectively. (**B**) Left, schematic showing the domain organization of TbRGG2 and the constructs analyzed in this study. Right, crystal structure of the TbRGG2 RRM domain with secondary structural elements and loops labeled. Strands, helices and loops are colored magenta, cyan and tan, respectively. Highlighted in red are the residues (RNP1 and RNP2 motifs) that are involved in RNA binding by canonical RRM domains. This and all cartoon and ribbon diagram figures were made using Pymol (60). (**C**) Size exclusion chromatography (SEC) analyses of the TbRGG2 RRM domain showing it elutes as a monomer. The y-axis is elution volume normalized for the column volume and the x-axis is log(MW). (**D**) Sequence of the TbRGG2 domain with secondary structural elements indicated and colored as in the cartoon in Figure 1B. Below is an alignment of the TbRGG2 RRM domain with RRM consensus RNP1 and RNP2 regions showing that none of the key aromatic residues involved in RNA binding by canonical RRM domains are conserved in the TbRGG2 RRM.

Previous studies showed that the TbRGG2 RRM can interact with various mRNA fragments (30). However, whether this domain displays RNA binding preferences is not known. As data indicates that stretches of repetitive RNA sequences are important for the kRNA editing process, we assessed the ability of the TbRGG2 RRM to interact with U20, C20, A20 and G20 RNA sequences. Interestingly, the data show that the RRM interacts with high affinity to U20 and G20 sequences, which it binds with *K*_d_s of 1.4 ± 0.03 μM and 27 ± 11 nM, respectively (Supplemental Figure S2). By contrast, the RRM interacted only weakly with A20 RNA (*K*_d_ = 6.3 ± 1.4 μM) and showed no measurable binding to C20 RNA (Supplemental Figure S2) (Table 2). Like the TbRGG2 RRM, most RRMs bind nucleic acid substrates with affinities in the nM to low μM range (36–37). However, TbRGG2 does not contain the signature aromatic residues within its predicted RRM RNA binding motifs, raising the possibility that it may not adopt a canonical RRM fold. To address this question we determined crystal structures of the domain, which encompasses residues 203 to 269, from the *T. brucei* protein (Figure 1A). Two apo RRM structures were obtained, one of the RRM(L209M) mutant, which was constructed to aid in crystallographic phasing (Materials and Methods) and one of the WT RRM. Both structures were solved to 1.8 Å to final *R*_work_/*R*_free_ values of 15.1%/18.7% and 16.5%/19.7%, respectively (Table 1).

**Table 2.** RNA binding affinities determined by fluorescence polarization

Protein	Poly(G)	Poly(U)	Poly(A)	Poly(C)	Telo GQ	DeazaG GQ	M1G GQ
RRM	27 ± 11 nM	1.4 ± 0.03 μM	6.3 ± 1.4 μM	No binding	ND	ND	ND
RRM(W215A)	50 ± 4 nM	No binding	ND	ND	ND	ND	ND
RRM(F230A)	39 ± 4 nM	No binding	ND	ND	ND	ND	ND
RRM(R203A)	157 ± 39 nM	ND	ND	ND	ND	ND	ND
RRM(R223A)	442 ± 43 nM	ND	ND	ND	ND	ND	ND
RRM(238A)	16 ±4 nM	ND	ND	ND	ND	ND	ND
RRM(R251A)	11 ± 3 nM	ND	ND	ND	ND	ND	ND
RRM(Q205A)	21 ± 4 nM	ND	ND	ND	ND	ND	ND
RRM-Cterm	1.9± 0.7 nM	240 ± 37 nM	1.0 ± 0.1 μM	2.6 ± 0.4 μM	28 ± 6 nM	156 ± 24 nM	163 ± 47nM
RRM-Cterm(W215A)	ND	No binding	ND	ND	ND	ND	ND
RRM-Cterm(F230A)	ND	No binding	ND	ND	ND	ND	ND

ND = Not determined.

The WT RRM structure contains four subunits in the crystallographic asymmetric unit (ASU) and the RRM(L209M) structure contains one. The RRM molecules in the two structures are essentially identical; the Cα atoms of the RRM in both structures can be superimposed with root mean square deviations (rmsds) of 0.4–0.6 Å. The structure shows that the TbRGG2 RRM indeed harbors the characteristic RRM fold composed of a five-stranded anti-parallel β-sheet surrounded by two α-helices (36–37) (Figure 1B). Structural homology analyses show that the TbRGG2 RRM domain exhibits structural homology to several RRM structures, in particular the RRM domains of the nuclear cap binding protein (1H6K) (44) and the human raver1 protein (3H2V) (45) with which the TbRGG2 RRM domain can be superimposed with rmsds of 0.77 and 0.78 Å for 66 corresponding Cα atoms. The TbRGG2 RRM structures revealed no interfaces significant enough to provide stable oligomerization, suggesting the domain itself does not oligomerize. Consistent with this, size exclusion chromatography (SEC) experiments revealed that the RRM domain, even at high concentrations (1 mM), elutes as a monomer (Figure 1C).

Although the TbRGG2 RRM adopts an RRM fold, as noted it does not contain the conserved aromatic residues located on the RNP motifs, characteristic of canonical RRM proteins. Instead it harbors valine, arginine and glutamine residues in these positions (Figure 1B, D). The structures of most RRM-nucleic acid complexes that have been solved reveal a classical mode of binding whereby 3–4 RNA or DNA nucleotides are bound across the β-sheet face of the domain. These nucleotides interact with aromatic residues on RNP1, which is located on β3, and RNP2, located on β1 (Figure 1D) (36–37). In cases where the nucleotide substrate is longer than ∼4 nucleotides, residues outside the RNP motifs have been observed to interact with the additional nucleotides (46–47). Thus, these and other RRM-RNA or RRM-DNA structures indicate that the RNP motifs serve as the main binding platform for the nucleic acids with adjacent regions augmenting interactions with longer substrates. Consistent with this finding, mutation of RNP aromatic residues in canonical RRMs significantly impairs nucleic acid binding by these proteins (36–37).

Exceptions to the canonical mode of RRM binding are two subclasses of RRM proteins called pseudoRRMs (48) and quasi-RRMs (qRRM) (49). RRMs of these subclasses do not contain conserved RNP residues. PseudoRRMs are instead defined by a sequence motif located on α1, SWQDLKD, which was shown to specifically recognize an RNA substrate in the SRSF1 RRM-RNA structure (48). The qRRMs of the heterogeneous nuclear ribonucleoprotein (hnRNP) F protein were shown to interact with RNA substrates using residues primarily located on loops 1 and 5, which contain conserved motifs, RGLP(W/F/Y) and (R/K)(X_5_)RY(V/I/L)F, respectively (49). The TbRGG2 RRM contains neither the canonical RNP residues nor the RNA binding residues that characterize qRRMs. Interestingly, however, it does harbor a tryptophan, Trp215, similarly positioned to the one in the pseudoRRM motif. But the rest of the pseudoRRM motif is not well conserved in the TbRGG2 RRM. Thus, it appeared possible that the TbRGG2 RRM may employ an RNA binding mode distinct from previously characterized RRMs.

### Structure of TbRGG2 RRM-U4 RNA complex

**Figure 2.**
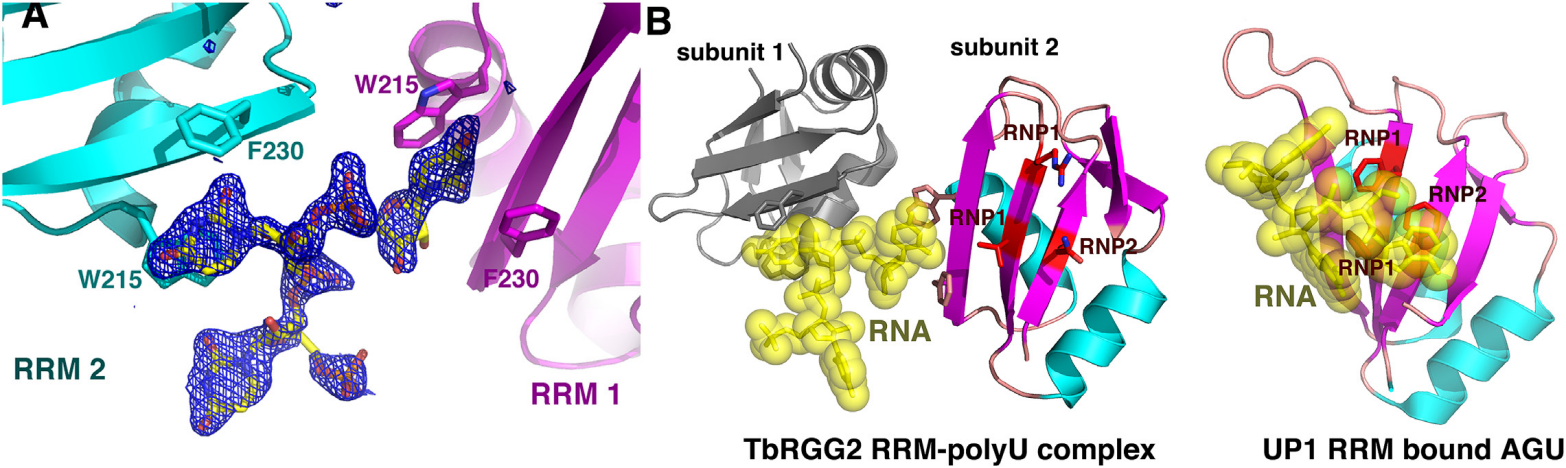
Molecular basis for U4 RNA binding by the TbRGG2 RRM domain. (**A**) Close up of the RNA binding pocket located between two RRM domains, one colored magenta and the other, cyan. The RNA is shown as sticks and the Fo-Fc electron density map calculated before addition of the RNA is shown as a blue mesh and contoured at 3.5σ. (**B**) Comparison of U4 RNA binding by TbRGG2 and RNA binding by a canonical RRM domain. Left, structure of the TbRGG2-U4 RNA structure. One RRM domain is colored as in Figure 1B and the other is gray. The RNP1 and RNP2 residues mediating RNA binding by canonical RRM domains are shown in red. The RNA is shown as yellow sticks with transparent surface. Right, overall structure of the UP1 RRM bound to RNA showing how RNP1 and RNP2 residues bind RNA in canonical RRMs.

**Figure 3.**
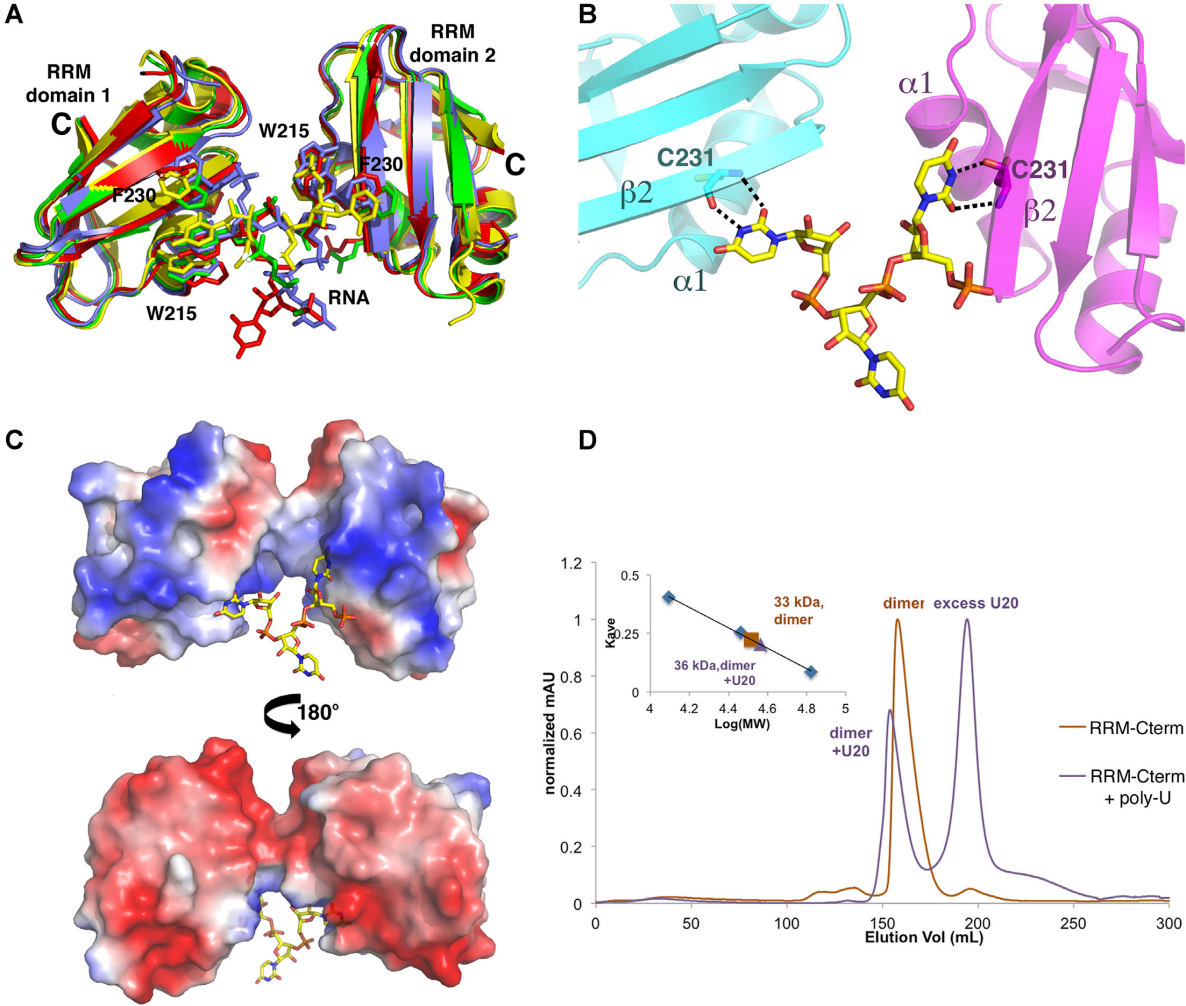
Mechanism of uridine RNA recognition by the TbRGG2 RRM domain. (**A**) Overlay of the distinct RRM-RNA complexes in the crystallographic ASU showing the same overall binding mode involving an RRM dimer. (**B**) Close up of uridine recognition by the TbRGG2 RRM domains. Each uridine N3 and O2 atom is read by the backbone carbonyl and amide nitrogen of Cys231, respectively. (**C**) Electrostatic surface representation of the two RRMs binding to U4 where red and blue represent electronegative and electropositive regions, respectively. (**D**) SEC experiments analyzing the RRM-Cterm with and without U20 RNA. The RRM-Cterm alone (orange chromatogram) runs at a molecular weight (MW) of 33 kDa and when U20 is present, the largest peak (purple) corresponds to a MW of 36 kDa, which is consistent with an RRM-Cterm dimer bound to one strand of RNA (RRM-Cterm dimer MW = 31 kDa and U20 MW = 6 kDa). The inset is the curve used to determine MW, where the y-axis is elution volume normalized for the column volume and the x-axis is log(MW).

To determine how the TbRGG2 RRM domain binds RNA, we obtained the structure of the domain bound to a poly-uridine (U4) RNA. The structure was solved by molecular replacement and refined to final *R*_work_/*R*_free_ values of 18.1%/22.9% to 1.95 Å resolution (Materials and Methods) (Table 1) (Figure 2A). Comparison of the RNA-bound RRM domains with that of the apo RRM structure shows no significant structural changes take place upon U4 RNA binding. The TbRGG2-RNA structure has a complex asymmetric unit. However, four similar TbRGG2 RNA complexes were revealed. In each of these complexes two RRMs combine to simultaneously contact one RNA using a unique binding mode distinct from that observed in other RRM–RNA complexes (Figures 2B and 3A). In the TbRGG2-RNA complex, key RNA base stacking interactions are provided by Phe230 from β-strand 2 and Trp215 from the N-terminus of α-helix 1, from two RRM subunits. Notably, Trp215 and Phe230 are completely conserved among TbRGG2 homologs (Figure 1A). In this novel RRM-RNA interaction, binding by the two RRMs splays apart the U4 substrate, suggesting a mechanism by which the protein may mediate melting or stabilization of single stranded RNA U stretches.

As noted, Trp215 is analogous to the tryptophan in the pseudoRRM motif that was shown to bind RNA in the SRSF1-RNA structure (48). However, the tryptophans make very different interactions with their RNA substrates in the two structures (Supplemental S3A). Also, because the TbRGG2 RRMs bind as a dimer, there are multiple clashes between the N-terminal region of the monomeric SRSF1 protein and its bound RNA with the TbRGG2 complexed RNA (Supplemental Figure S3A). The TbRGG2 RRM RNA binding mode is also distinct from that of hnRNP F; TbRGG2 binds U4 between the two subunits far from the loops employed by hnRNP F to bind RNA (Supplemental Figure S3B). Moreover, loops 1 and 5 also adopt very different conformations in two proteins (Supplemental Figure S3B). In the TbRGG2 RRM-U4 structure, the uridine O2 and N3 atoms are read by the backbone amide nitrogen and carbonyl oxygen of RRM residue Cys231 (Figure 3B). Because backbone atoms are more rigid than side chains, they provide strong selectivity in ligand binding. Thus, this recognition mode would appear to select against binding guanine, adenine and cytosine containing nucleotides; a cytosine would place a hydrogen bond donor (N2) next to a hydrogen bond donor (the Cys231 NH amide group), while adenine and guanine bases would not only result in unfavorable hydrogen bonds but also clashes with the backbone of Cys231 (Supplemental Figure S4).

In addition to the RNA contacts from the Cys231 backbone and the side chains of Phe230 and Trp215, several basic residues either make contacts to the RNA phosphate backbone (Lys219) or are positioned to provide electrostatic contacts to the RNA, such as Lys230. In fact, examination of the electrostatic surface representation of the two RRM subunits interacting with the RNA reveals that the face of the dimer that contacts the RNA is notably electropositive (Figure 3C).

### A region C-terminal to RRM mediates TbRGG2 dimerization

**Figure 4.**
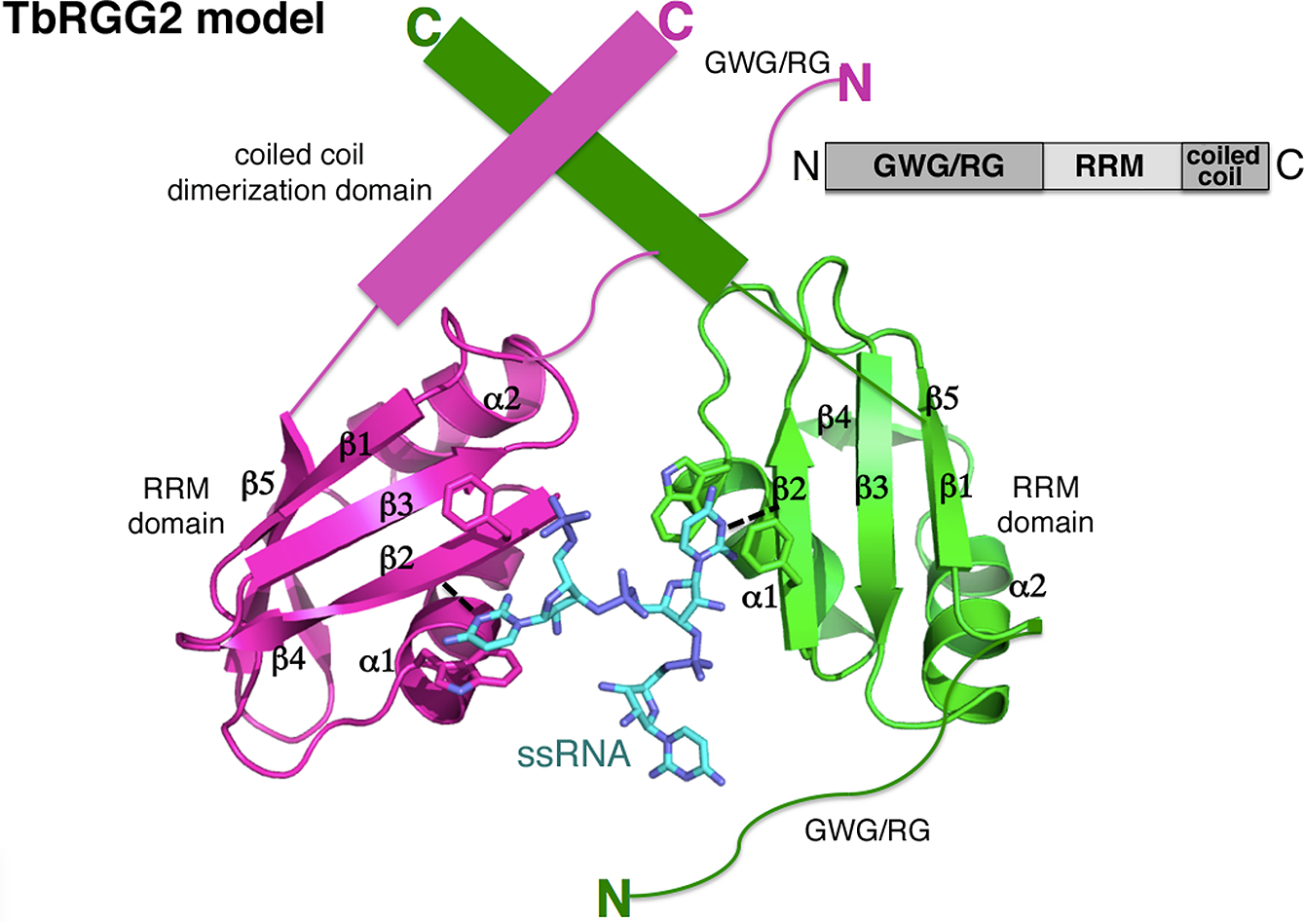
Structure and biochemical based model for the full length TbRGG2 protein showing it in complex with U4 RNA. One subunit of the TbRGG2 dimer is colored magenta and the other green. The bound RNA is cyan. Also shown are key stacking residues that contact the U4 substrate. Upper right is a schematic of the TbRGG2 domain organization.

The TbRGG2-RNA structure revealed that the TbRGG2 RRM binds U4 RNA as a dimer. However, the TbRGG2 RRM domain itself is not dimeric and there is currently no information as to the oligomeric state of full length TbRGG2. Gly-rich regions such as the N-terminal domain of TbRGG2 are primarily disordered in the absence of substrates and not known to oligomerize. Moreover, the Gly-rich domain of TbRGG2 orthologs are not conserved in sequence or length as might be expected for an oligomerization region (Figure 1A). Sequence alignments, however, show two regions, in addition to the RRM, that are highly conserved between TbRGG2 orthologs, one N- terminal and one C-terminal to the RRM domain (Figure 1A). The N-terminal region contains multiple prolines, glycines and serines and is predicted to be flexible or disordered, similar to the Gly-rich domain. By contrast, the region C-terminal to the RRM, residues 270–320, is strongly predicted to form a coiled coil. As coiled coils are often utilized in forming oligomers, we generated a construct encoding TbRGG2 residues 203–320 (RRM-Cterm), produced purified protein and performed SEC analyses to analyze its oligomeric state. The SEC results showed that this protein, unlike the RRM domain alone, is dimeric (Figure 3D). Moreover, when we performed SEC on the RRM–Cterm–U20 RNA complex we obtained a molecular weight consistent with a dimeric RRM–Cterm and a single RNA molecule (Figure 3D). Thus, the combined data suggest a model in which TbRGG2 residues 270–320 form a dimerization module, which would juxtapose the RRM domains allowing them to bind an RNA molecule (Figure 4).

### Probing the structural model via fluorescence polarization

**Figure 5.**
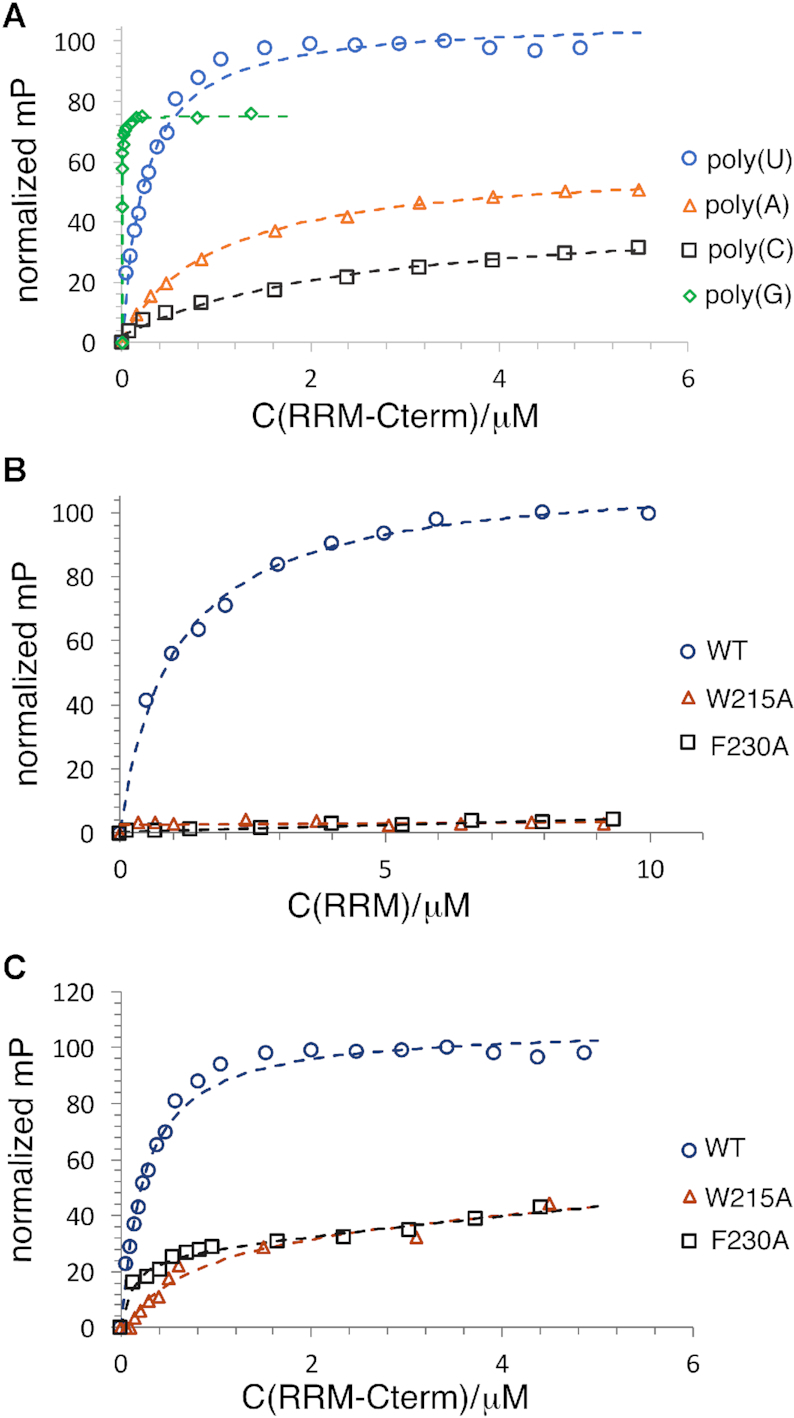
FP studies revealing TbRGG2 RRM RNA binding enhancement by dimerization. (**A**) FP binding isotherms of the TbRGG2 RRM binding to 20-mer RNA fragments, either U20 (blue circle), A20 (orange triangle), C20 (black square) G20 (green diamond). Each binding curve from this and all FP curves, is a representative curve from three technical replicates. The y-axis is the normalized mP (×100) and the x-axis is the concentration of TbRGG2 construct (as indicated by C(RRM or RRM–Cterm)/μM). (**B**) FP binding isotherms of U20 binding to the WT TbRGG2 RRM (blue circle), the W215A RRM mutant (orange triangle) and the F230A RRM mutant (black square). (**C**) The same binding analyses as 5B but using the TbRGG2 RRM-Cterm protein; U20 binding to the WT RRM-Cterm (blue circle), the W215A RRM–Cterm mutant (orange triangle) and the F230A RRM–Cterm mutant (black square).

Previous studies on dimeric RNA and DNA binding proteins have shown that dimerization domains, even if not contributing directly to nucleic acid binding, lead to enhanced binding (50–51). For example, studies on DNA binding proteins, including prokaryotic response regulators and DNA segregation proteins, and multiple eukaryotic proteins such as STAT proteins that contain DNA binding domains attached to dimerization domains, show that generation of the dimer leads to higher affinity DNA binding and in some cases is required for measurable DNA binding (50–51). If this is case for TbRGG2, the expectation would be that the RRM-Cterm should display higher affinity binding to RNA compared to the RRM domain alone, but also show the same preference for U20 and G20 RNA. To test this hypothesis, we employed FP binding assays with the TbRGG2 RRM–Cterm protein. The data showed that this construct bound RNA with significantly enhanced affinities compared to the RRM domain alone and with the same preference. Specifically, U20, G20, A20 and C20 were bound with *K*_d_s of 240 ± 37 nM compared to 1.4 μM, 1.9 ± 0.7 nM compared to 27 nM, 1.0 ± 0.1 μM compared to 6.3 μM and 2.6 ± 0.4 μM compared to no binding, respectively (Figure 5A) (Table 2). Thus, the FP analyses support the structural data suggesting that the TbRGG2 RRM interacts with RNA as a dimer. The RRM-U4 structure also indicates that residues Phe230 and Trp215 would be critical for poly(U) RNA binding. To test this prediction, FP analyses were carried out on W215A and F230A RRM mutant proteins. The results show that these mutations essentially abrogated poly(U) RNA binding by the TbRGG2 RRM and RRM-Cterm proteins (Figure 5B, C).

### TbRGG2 RRM binds to poly(G) and poly(U) using different surfaces

**Figure 6.**
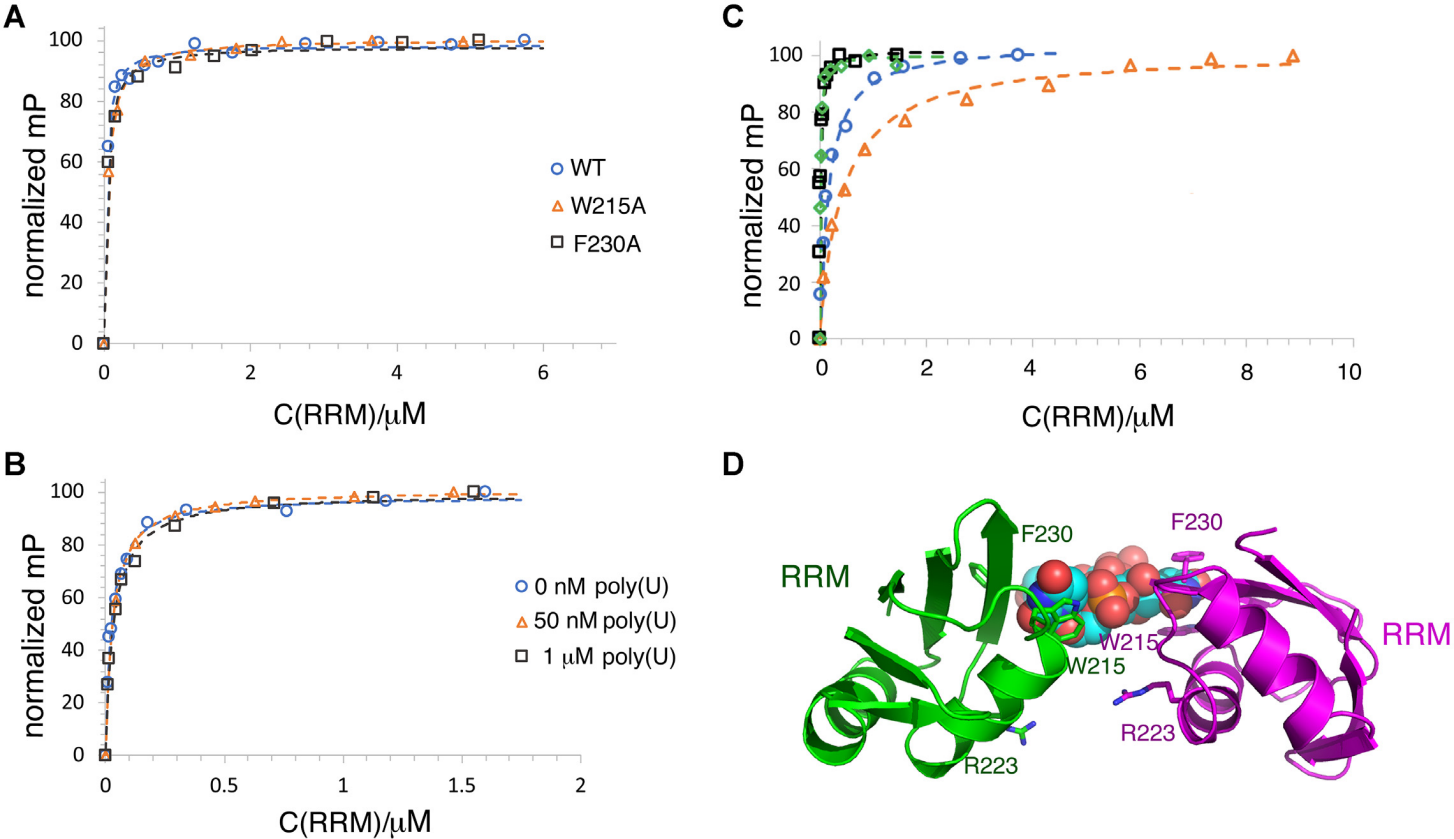
The TbRGG2 RRM binds poly(G) and poly(U) using different binding sites. (**A**) FP binding curves showing that WT (blue circle), W215A (orange triangle) and F230A (black square) TbRGG2 RRMs bind to G20 RNA with essentially the same affinity (*K*_d_s are 27 ± 11, 50 ± 4 and 39 ± 4 nM, respectively). (**B**) FP binding isotherm analyzing binding of G20 RNA to free RRM (blue circle), RRM prebound to 50 nM U20 RNA (orange triangle) and RRM prebound to 1 μM U20 RNA (black square). The axes are labeled as in Figure 5. The *K*_d_s for the latter are 26 ± 0.7, 31 ± 6 and 36 ± 6 nM, respectively. (**C**) FP analysis of G20 binding to RRM mutants, R203A (blue circle), R223A (orange triangle), R238A (black square) and R251A (green diamond). The *K*_d_s are 157 ± 39, 442 ± 43, 16 ± 4 and 11 ± 3 nM, respectively. (**D**) Ribbon diagram showing the locations of the arginines on the RRM dimer-U4 structure that were mutated to assess the effects on G20 binding. The uridines are shown as spheres and the arginines as sticks. Notably, mutating Arg223 had a significant impact on G20 binding but is located on the face opposite where poly(U) binds.

Our FP studies revealed that the RRM domain binds with high affinity to U20 and G20 RNA. Interestingly, however, the RRM bound with significantly higher affinity to poly(G) sequences than poly(U) RNA (Supplemental Figure S2; Figure 5A). These data and the fact that the RRM-U4 structure revealed what appeared to be specific interactions between the protein backbone and the uridine bases, suggested that the RRM might bind poly(G) in a manner distinct from poly(U). To investigate this idea we analyzed G20 binding by the RRM(W215A) and RRM(F230A) mutant proteins. FP analyses showed that these mutants bound G20 with essentially the same affinity as the WT RRM (Figure 6A) indicating that the TbRGG2 RRM interacts with poly(G) using a different surface than that used to bind poly(U). Such a multimodal binding capability would predict that the RRM should be capable of binding both U20 and G20 RNAs simultaneously. To test this hypothesis, we analyzed G20 binding to the RRM that was pre-bound to excess U20. These experiments showed that, indeed, G20 bound with essentially the same affinity to an RRM solution with pre-bound U20 as to the RRM in the absence of U20 (Figure 6B).

Though the TbRGG2 RRM does not employ RNP residues for poly(U) binding, the possibility existed that it may use these residues to bind poly(G) even though they do not contain the conserved aromatic residues found in canonical RRM domains. To address this possibility we generated Q205A and R238A mutations in the TbRGG2 RRM and examined G20 binding. These experiments showed that these substitutions had no significant effect on G20 binding (Table 2) indicating that G20 binding does not occur on the TbRGG2 RRM β-sheet face (Supplemental Figure S5; Figure 6C). Guanine bases are often recognized by arginine residues. The R238A mutation had no effect on binding so we next generated arginine to alanine mutations in the remaining arginine residues of the RRM and analyzed G20 binding. While the R203A substitution had an effect on binding, only the R223A resulted in a significant decrease (16-fold) in G20 binding (Figure 6C). This arginine, which is conserved in all TbRGG2 homologs, is located adjacent to the poly(U) binding site but on the opposite face of the RRM domain dimer (Figure 6D). Thus, our combined data suggest that the TbRGG2 RRM binds poly(U) and poly(G) RNA substrates using distinct binding surfaces.

### Mechanistic insight into RRM poly(G) binding

**Figure 7.**
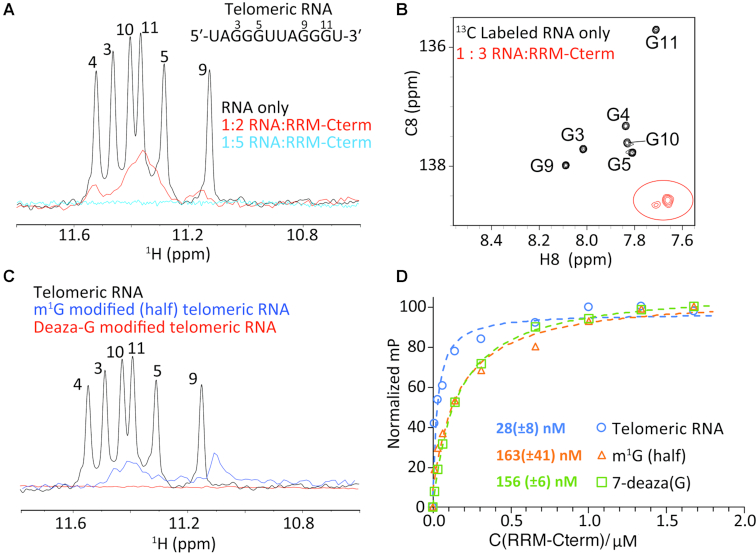
TbRGG2 RRM binds single stranded guanine stretches. (**A**) 1H NMR spectra of the imino region of the RNA, 5′-UAGGGUUAGGGU-3′ in the absence (black) and the presence of TbRGG2 RRM-Cterm at RNA:protein ratios of 1:2 (red) and 1:5 (blue). (**B**) 2D aromatic spectra of G-C8 15C labeled telomeric RNA in the absence (black) and presence (red) of RRM-Cterm protein. (**C**) NMR spectra corresponding to the imino regions of the WT (black), m1G (blue) and 7-deaza (red) RNAs, showing that the latter 2 RNAs are not structured. (**D**) FP analyses of the TbRGG2 RRM binding to WT 5′-UAGGGUUAGGGU-3′ RNA (blue), the m1G substrate in which the same RNA was used but with the first three guanines substituted with m1G (orange) and 7-deaza in which all the guanines were replaced with 7-deazaguanine (green). The resultant *K*_d_s were 28 ± 8, 163 ± 41 and 156 ± 6 nM, respectively.

Poly(G) RNA repeat elements like the ones we utilized in binding studies with the TbRGG2 RRM are predicted to form higher order structures, in particular G-quadruplex arrangements (52). This finding was interesting in light of a recent study that revealed that a characteristic feature of kinetoplastid pan-edited pre-mRNA transcripts is the presence of numerous quadruplex forming guanine clusters (14–15). The kRNA editing accessory protein, MRB1590 was suggested as a possible G-quadruplex resolving factor (14,32). However, we recently showed that MRB1590 acts primarily at one specific G-rich pause site located in the A6 pre-mRNA transcript, suggesting it may not function as a general G-quadruplex resolving factor (14,32). By contrast, TbRGG2 is known to be essential for the successful editing of all pan-edited transcripts (28–29). Our finding that it binds G20 with high affinity thus raised the question as to whether the RRM was binding the poly(G) substrates as quadruplexes or as single stranded nucleotides. To address this issue in more detail we performed binding studies on a well characterized, quadruplex forming telomeric RNA repeat, 5′-UAGGGUUAGGGU-3′ (53–54). This 12 nucleotide human telomeric RNA was previously shown to form an intermolecular, propeller-type G-quadruplex with three G-tetrad layers (52–53). And while the quadruplex forming regions in the trypanosome mRNA are intramolecular, the overall folds are similar and hence this RNA may serve as a model for such regions. FP analyses revealed that the TbRGG2 RRM-Cterm bound this RNA similar to G20 RNA, with a *K*_d_ of 28 ± 6 nM (Supplemental Figure S6). We next used NMR to assay the affect of the TbRGG2 RRM on telomeric RNA structure. The 1D NMR spectrum obtained of the RNA alone revealed significant signals (at ∼11 ppm) that are characteristic of Hoosgteen base pairing, as expected for quadruplex structures. These signature imino resonances gradually disappeared out of detection with added TbRGG2 RRM-Cterm protein (Figure 7A). The disappearance of the imino resonances is consistent with melting of the G-quadruplex to form single stranded RNA. Indeed, the 2D CH SOFAST-HMQC aromatic spectra (43) of G-C8 isotopically labeled RNA in the absence and presence of the RRM-Cterm dimer (Figure 7B and Supplemental Figure S7) revealed resonances that are downfield shifted in the carbon dimension, consistent with formation of single-stranded RNA (Figure 7B). These data help rule out generic broadening of resonances due to the formation of a large complex, which would be expected to broaden all resonances in the RNA.

The NMR data suggest that the TbRGG2 RRM may promote melting of Hoogsteen pairing and possibly the entire G-quadruplex structure. To further test this hypothesis, we replaced guanines in the sequence with 7-deazaguanine and *N*1-methylguanosine (m1G). These RNA substrates do not form quadruplexes as they cannot make stable Hoogsteen base pairs. This was confirmed by 1D NMR analyses of each substrate (Figure 7C). Hence, if the TbRGG2 RRM specifically binds quadruplex structures, the expectation is that it would not interact with these RNAs. However, if the RRM melts G-quadruplexes and stabilizes unfolded guanine RNA, binding to these substrates should be observed with the caveat that the affinity may be weaker if some of the substituted atoms are involved in TbRGG2 binding. For the 7-deaza substrate, all the guanines in the quadruplex-forming sequence, 5′-UAGGGUUAGGGU-3′, were replaced with 7-deazaguanine and for the m1G substrate, only the first 3 guanines were modified. The FP experiments showed that while the affinities were reduced, the RRM indeed interacted with these modified RNA substrates with high affinity (Figure 7D). Hence, our combined NMR and FP data suggest that the TbRGG2 RRM is able to bind and stabilize single stranded guanine stretches. However, further experiments are needed to deduce the specific poly(G) binding mechanism utilized by the protein.

## DISCUSSION

Kinetoplastid protists undergo a unique form of RNA editing in which uridines are inserted and deleted to create translatable mitochondrial mRNA transcripts. In the most extensively edited transcripts, called pan-edited transcripts, this RNA modification can result in up to 60% of the final mRNA being derived from editing (3–7,13). Although the editosome machinery catalyzes the insertion/deletion steps, it is not sufficient to obtain a mature mRNA transcript and a diverse set of accessory factors have been identified as also necessary for the process. Detailed structural and biochemical analyses have been performed, however, for only a handful of these proteins. And the proteins studied thus far mediate editing for only one or a few transcripts. By contrast, the TbRGG2 accessory factor is required for the editing all pan-edited transcripts (28–29). TbRGG2 is required for all stages of the parasite's life cycle, but its protein levels are up-regulated 10-fold in procyclic forms of the parasite compared to the bloodstream stage. This up-regulation is consistent with the central role of TbRGG2 in kRNA editing as this process generates mRNAs encoding proteins involved in oxidative phosphorylation, which takes place in the procyclic form, whereas in the mammalian bloodstream form, energy can be obtained through glycolysis. The TbRGG2 protein contains two putative RNA binding motifs, an N-terminal Gly-rich region, and a putative RRM domain, both of which were shown to be essential for its pan-editing functions (28–30).

Here, we show that the TbRGG2 RRM domain specifically recognizes and modulates the structure of poly(U) and poly(G) RNA. A previous study using full length TbRGG2 protein as a GST fusion construct (GST-TbRGG2) also showed poly(U) binding (29). However, GST itself is known to dimerize, possibly complicating these results; indeed we showed that TbRGG2 dimerizes via a C-terminal conserved region and that this specific dimerization effects RNA binding affinity. Previous studies suggested that the intrinsically disordered, Gly-rich N-terminal region of TbRGG2 may function in annealing (30) and hence could collaborate with the RRM, which we showed unfolds RNA stretches, in some still unknown manner to facilitate the editing process. Indeed, our data on the RRM show that it binds and unfolds specifically poly(U) and poly(G) RNA stretches, both of which are present in the mitochondrial mRNAs. In particular, recent data revealed that guanine stretches are specifically clustered in pan-edited transcripts; a study by Leeder *et al.* showed that 67% of guanines in pre-edited mRNAs are arranged in clusters but after editing, G-tracts are reduced to 25% (14–15). Our data indicate that the TbRGG2 RRM is able to unfold or stabilize the single stranded state of poly(U) and poly(G) RNA stretches. The TbRGG2 RRM-U4 structure showed that two RRM subunits of a TbRGG2 dimer encase the RNA as a single stranded form, in which Trp215 and Phe230 mediate base stacking contacts. Supporting the structural model, mutagenesis of the Trp215 and Phe230 to alanines abrogated U20 RNA binding. The apparent specificity in base recognition revealed by the RRM-U4 structure suggested that the protein might bind guanine repeats using a distinct mechanism. Our combined data showing that mutation of R223, located on the face opposite of the poly(U) binding site, impaired poly(G) binding while the poly(U) disrupting mutations, W215A and F230A did not disrupt poly(G) interactions, support that poly(G) and poly(U) are bound in different surfaces of the TbRGG2 RRM. Our combined fluorescence polarization and NMR data support the notion that the TbRGG2 RRM is able to melt or stabilize the single stranded form of guanine repeats.

Recent data have shown that OB-fold proteins, which dock on the surface of the editosome, harbor RNA chaperone activity and hence appear to play crucial roles in the editing process including, importantly, G-quadruplex unfolding (55). Our data on the TbRGG2 RRM domain suggest the possibility that it could also function as a G-quadruplex resolving factor. In fact, RRM containing proteins have been shown to constitute a major class of G-quadruplex binding and resolving proteins. In particular, the RRM domains of the hnRNP family of proteins (56–59). Structures show that the RRMs of hnRNP A1 and hnRNP D use canonical RNP contacts to stabilize single stranded guanine substrates while the hnRNP F RRM units employ residues in loops to bind single stranded guanine repeats (56–59). TbRGG2 uses yet a different region to interact with poly(G) stretches in a single stranded state. It is remarkable that the small RRM domain has evolved several different surfaces to selectively bind and stabilize single stranded guanine repeats.

In conclusion, our combined data have revealed that the RRM from the essential kRNA editing factor TbRGG2 utilizes a non-canonical mode of RNA binding by an RRM protein and furthermore, harbors multimodal binding capability allowing it to bind multiple RNA substrates on different surfaces. It shows a preference for binding RNA poly(U) and poly(G), which are repeats elements characteristic of the kRNA editing process. How the unique RNA binding and remodeling features of the TbRGG2 RRM are involved in kRNA editing remains to be determined.

## DATA AVAILABILITY

Coordinates and structure factor amplitudes for RRM(L209M), WT RRM and the WT RRM-U4 complex have been deposited with the Protein Data Bank under the Accession codes 6E4N, 6E4O and 6E4P.

## Supplementary Material

Supplementary Data
