# Supplementary material for: The RRM of the kRNA-editing protein TbRGG2 uses multiple surfaces to bind and remodel RNA
Source: Nucleic Acids Res. 2018 Dec 14;47(4):2130–42. doi: 10.1093/nar/gky1259 (PMC6393287; doi:10.1093/nar/gky1259)
Supplement: Supplementary Data [file gky1259_supplemental_files.pdf]

Figure S1

UGAUACAAAAAAACAUGACUACAUGAUAAGUAuCAuuuuAuGuuAuuuuu
GGuAGuuuuuuuACAuuuGuAuCGuuuuACAuuuG-GUCCACAGCAuCCCG-
--CAGCACAuG--GuGuuuuAuGuuGuuuAuuGuAuuuuuGuGGuGA-AuuuAu
uGuuuA--UAUUGAuUGuAuuAuA---GGuuAUUUGCAUCGUGGUACAGA
AAAGUUAUGUGAAUAUAAAAGUGUAGAACAAUGUCUUCCGuAUUUC
GACAGGUUAGAuuAuGuuA-GuGuuuGuuGuAAuGAGCAuuuGuuGuCuuu
A---UGuuuuGAGuAuAuGuuGCGAuGuuGuuuGuCGuuACGuuGuGCAuuuA
uuAAuuGuA----GAAuuuAC---CCGuAGuuuuAAuGGuuuGuuGuGuAuAuC
AuGuAuGGuuuuGG-AuuuAGGuuGuuuGuCUCCGuuG-UUAuGAuCAuuuG
AGGAA---CG-UGACAAAuuGAuGACAuuuuuuGAuuuAuG--UUGuGGuuG
uCGuAuGCAuuuGGCUUUCAuGGuuuuAuuA-GGuAUUCUUGAUGAuuuuG
uuuuuGGuuuuGuuGAuuuuuuGuuGuuGuuGA---UAAuAuCAuGuuuGuuuGu
uAuGGAuuGuuAuGAuuuGuuAuuuGuGGGuAAUCGuuuAuuuUAuuuGCGuu
uGC---GuGGuuuGuCAuuuuuuGAuuuAuAuGAuuuA--GuuuuuA--A--UAG
uuuAAGuGGuGuuuuGuCuCGuuCGuuAGGuAuGGuGuGAGAuuGUCGuuu
AuuuAGuuGuuA----UGA----GuUGuAuuuuAuGuuuuGuuAuGAuuAuuGuu
uuuGuuuuAuAGGuGAuGCAuuuGA-UCGuuuAuuuuuACGuuuGuuuGAUAu
GCGuAuGAGuuuGuuGAuuuGuAAGCAAuGuuuuuuuGuuGGuuuuuuuGuuu
uuG-----GuuuuGuuuGuuuGuuuG--AuuAuuuAuAuuGuGAuAuuACCAuuG-
---AGACCAuuAuuAuGuuAuuuuAuAGuuuGuGGuGuuGuuGuuuGCCGGGu
AuA-UCAuuuGC-UUGUGuuGAACACCCCAAAGGuGA---GuAuuGuuuGu
uAuuA----UGuuuuuGuGuuGGuuuAuGuuCUCGuuuACGuuuGCGuuGuGC
GGAuuuuuuGCA-UAUUUGuuuAuuGGAuGuuuGuuuGCGuGGuuuuuuAuuG
CAuGAuuuAGuuGC---C-GuuuuAGGuAAuAuuGAuGuuGuuuuuGGAuCCG
UAGAUCGuuA-GuuuuAuAuGuG--A------GGUUAUUGuAGGAUUGUUU
AAAAUUGAAUAAAAA-poly(A)

Figure S1. Sequence of the mature pan-edited ND7 mRNA from *T. brucei*. The red uridines are added by the editing process while the blue dashes represent positions were uridines are deleted. This figure was modified from the ND7 figure from http://dna.kdna.ucla.edu/parasite_course-old/RNA editing/subchapters/discovery_of_editing.html.

Figure S2

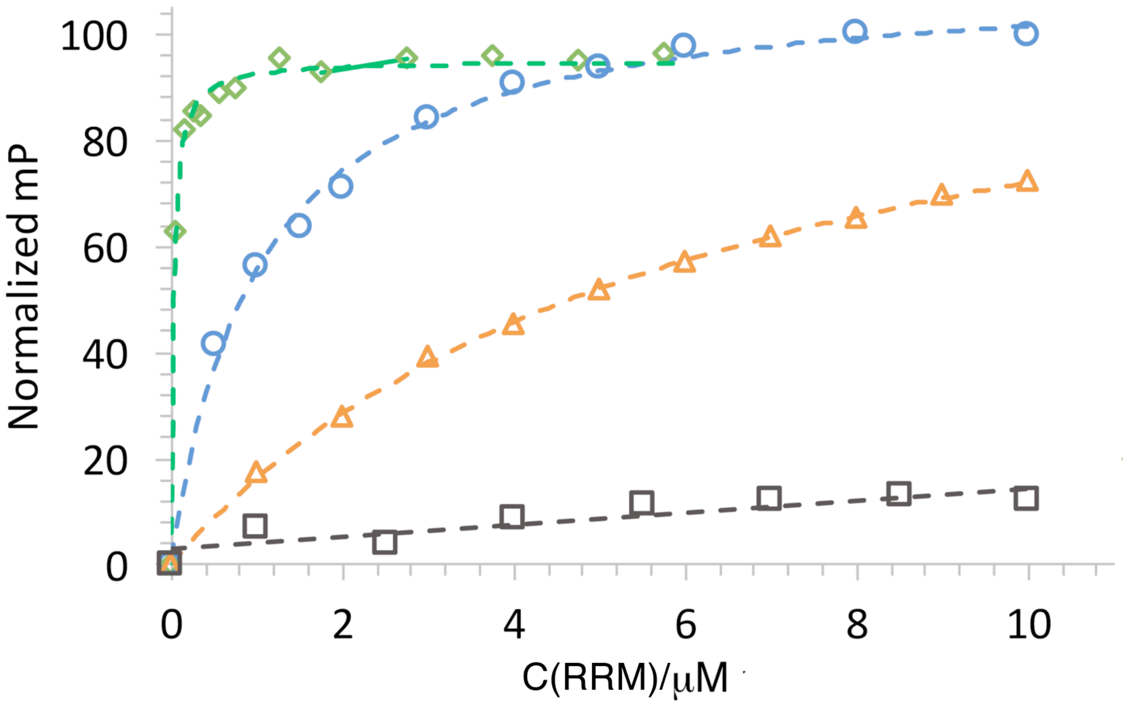

Figure S2. Fluorescence polarization (FP) binding studies analyzing TbRGG2 RRM RNA binding preference. Shown are FP binding isotherms of the TbRGG2 RRM binding to 20-mer RNA fragments consisting of either U20 (blue circle), A20 (orange triangle), C20 (black square) or G20 (green diamond). The x-axis is the RRM concentration in μM and the y-axis is the normalized mP.

Figure S3

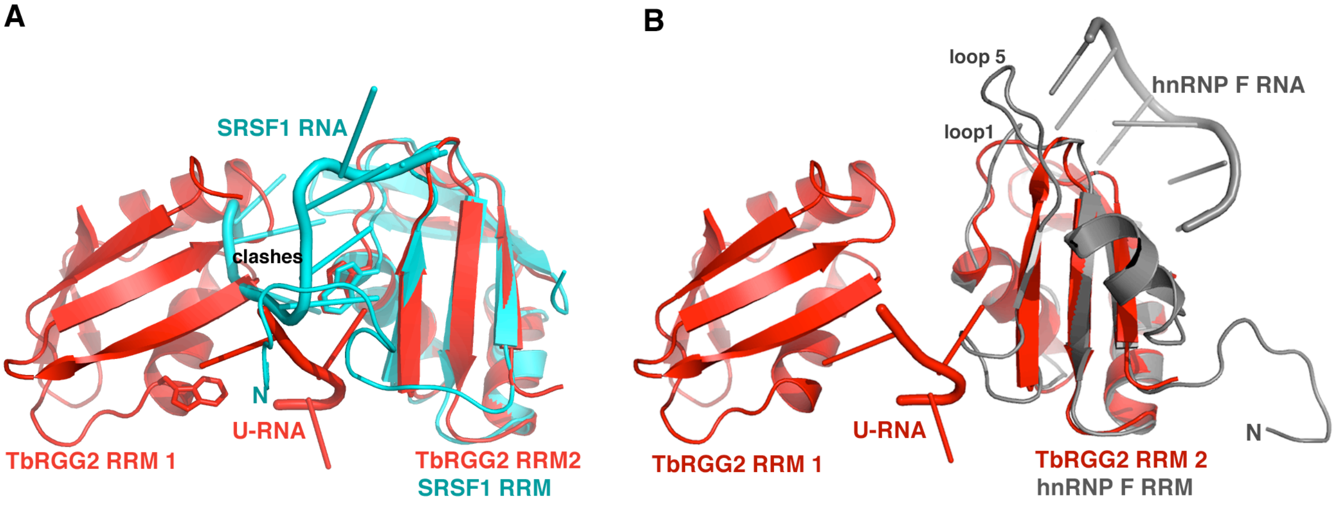

Figure S3. The TbRGG2 RRM-poly-U binding mode is distinct from pseudoRRM-RNA and qRRM-RNA binding modes. (**A)** Superimposition of one subunit of the TbRGG2-poly-U dimer (red) onto the RRM of the SRSF1-RNA (cyan) structure. The resulting rmsd for 66 Cα atoms is 1.1 Å. Shown as sticks are the tryptophan residues located at the N-terminus of helix 1 that are shared on both structures and used to stack with RNA bases. Note, however that the stacking interactions made by these tryptophans are different in TbRGG2 compared to SRSF1. In addition, the N-terminal region of SRSF1 clashes with the RNA bound by the TbRGG2 dimer and the position of the bound RNA in the SRSF1-RNA structure also clashes with the other RRM subunit of the TbRGG2 dimer. (**B**) Superimposition of one subunit of the TbRGG2-poly-U dimer (red) onto the RRM of the hnRNP F-RNA (grey) structure. The resulting rmsd for 63 corresponding Cα atoms is 1.5 Å. hnRNP F binds its target RNA using residues primarily from loops 1 and 5, which have different conformations and lengths compared to the corresponding loops in the TbRGG2 RRM. The RNA bound by hnRNP F is also docked on the opposite side of the RRM domain as is the RNA substrate in the TbRGG2-RNA structure.

Figure S4

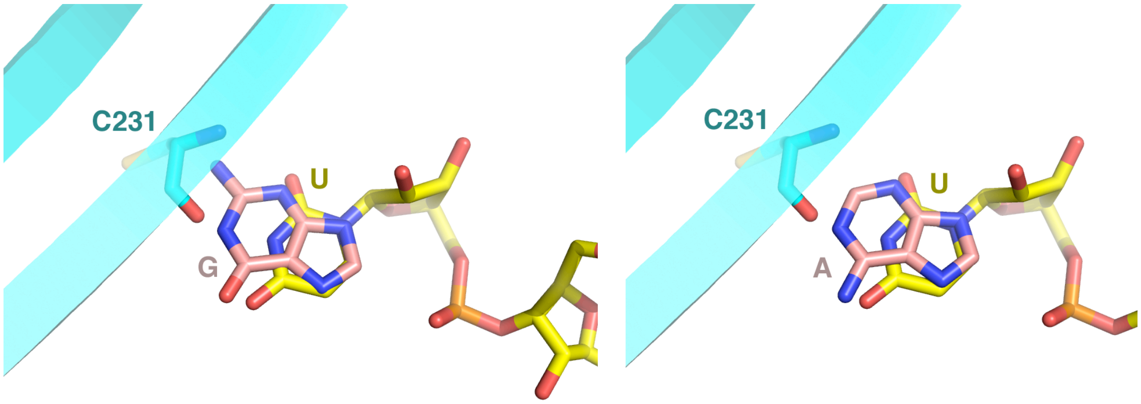

Guanine and adenines in place of uridine/ unfavorable H-bonds and clash

Figure S4. The TbRGG2 recognizes poly-U RNA via specific hydrogen bonds to backbone atoms. The TbRGG2-poly-U structure reveals specific hydrogen bonds between the uracil N3 and O2 atoms and the backbone carbonyl and amide nitrogen moieties of Cys231 on both RRM dimers. Modeling shows that adenine and guanine bases would not only be unable to make the same specific hydrogen bonds but would also clash with the RRM.

Figure S5

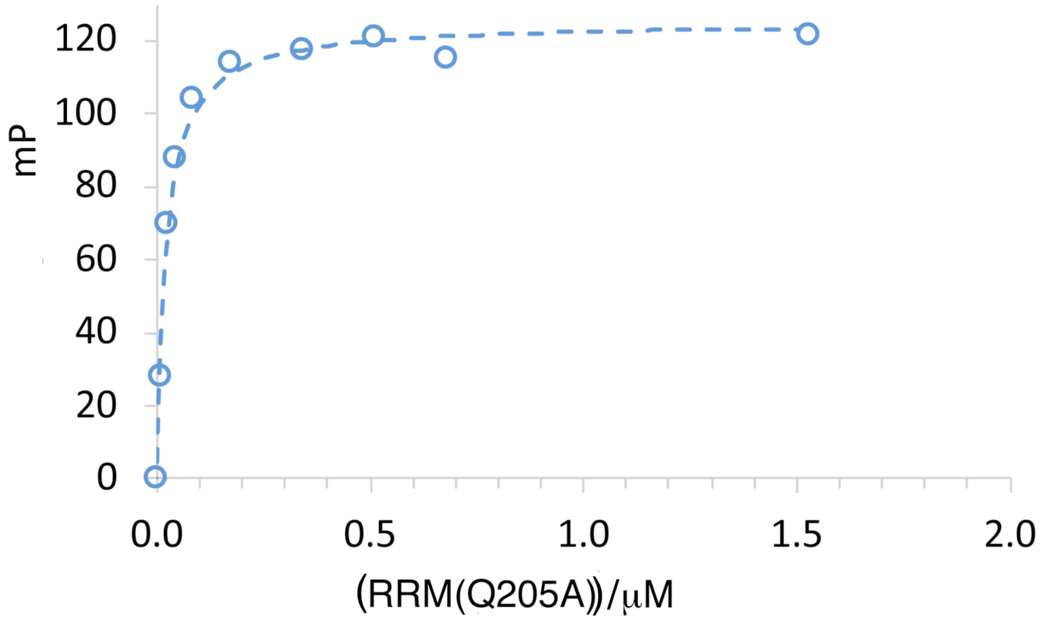

Figure S5. FP binding isotherm examining G20 binding by the TbRGG2 RRM(Q205A) mutant. The resultant K_d was 21 ± 4 nM, which is essentially the same as the value obtained by the binding isotherm of the WT RRM binding to G20. The x-axis is the RRM(Q205A) concentration in μM and the y-axis is in mP units.

Figure S6

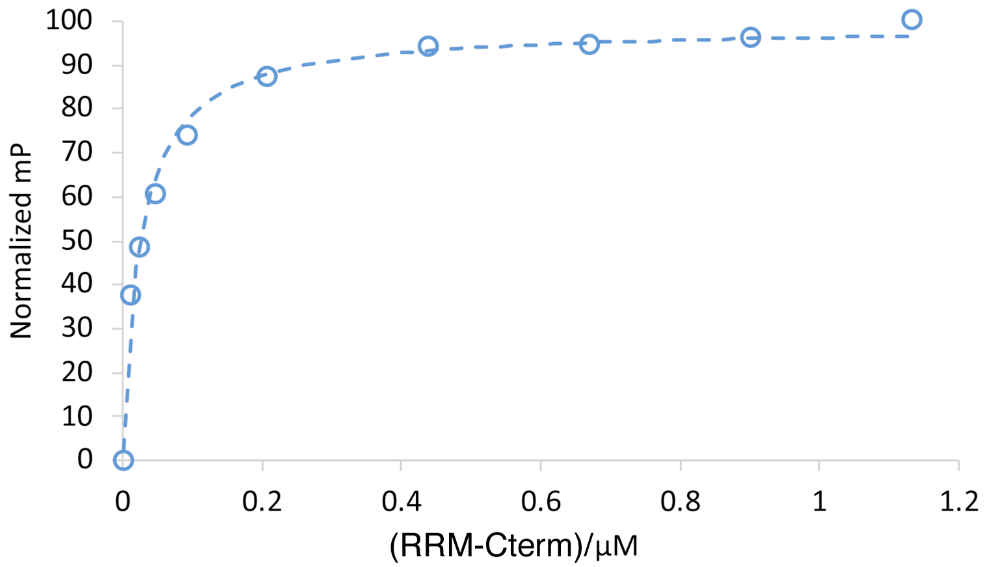

Figure S6. FP binding isotherm investigating binding to the RNA telomeric sequence by the TbRGG2 RRM-Cterm protein. The x-axis is the RRM-Cterm concentration in μM and the y-axis is normalized mP units.

Figure S7

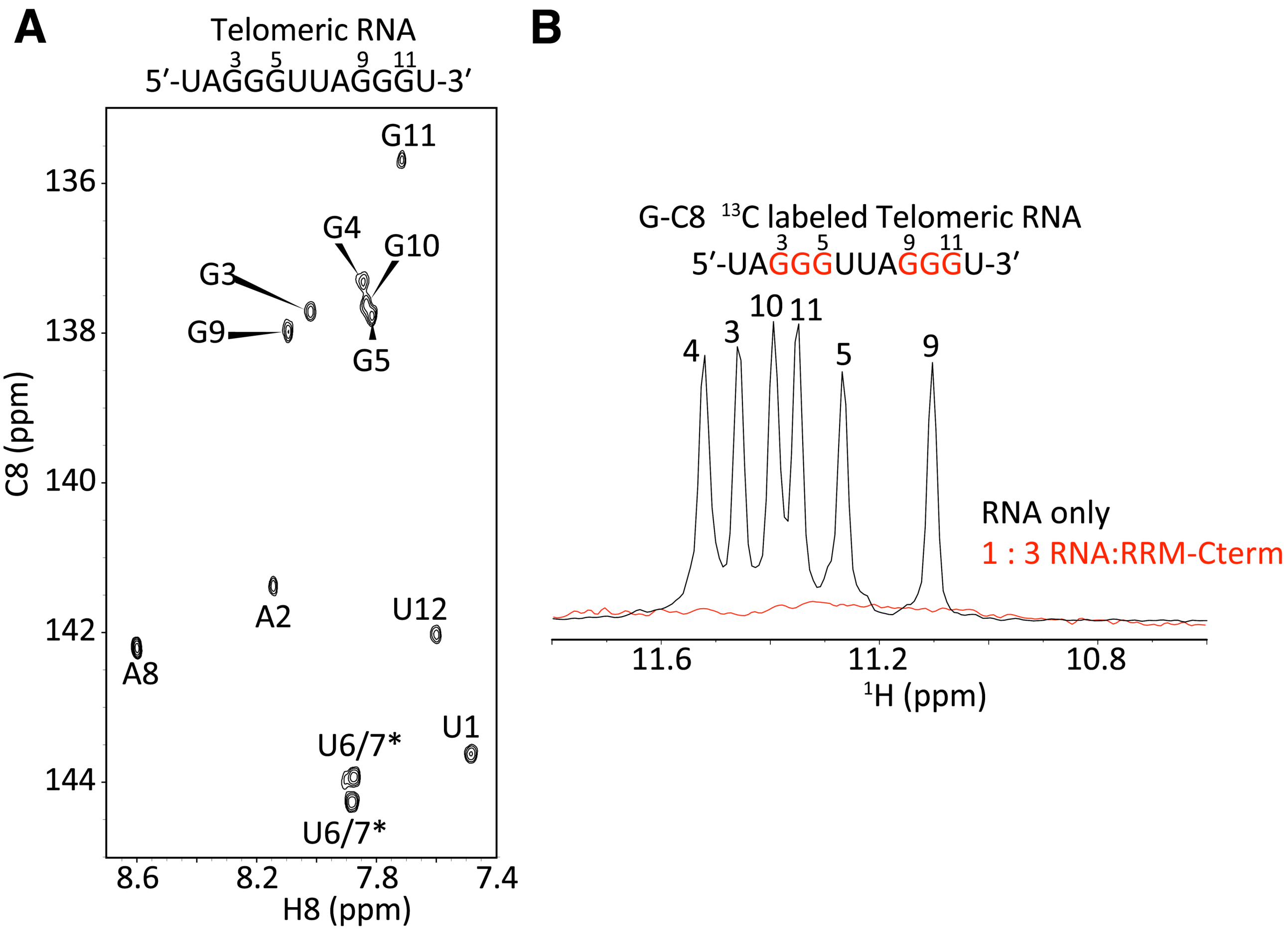

Figure S7. NMR spectra of telomeric RNA. (**A**) 2D aromatic spectra of telomeric RNA. (**B**) ^{1}H spectra of G-C8 ^{13}C labeled telomeric RNA in the absence (black) and presence (red) of RRM-Cterm protein.
